# Peroxynitrite Activated Drug Conjugate Systems Based on a Coumarin Scaffold Toward the Application of Theranostics

**DOI:** 10.3389/fchem.2019.00775

**Published:** 2019-12-05

**Authors:** Maria L. Odyniec, Hai-Hao Han, Jordan E. Gardiner, Adam C. Sedgwick, Xiao-Peng He, Steven D. Bull, Tony D. James

**Affiliations:** ^1^Department of Chemistry, University of Bath, Bath, United Kingdom; ^2^Key Laboratory for Advanced Materials and Feringa Nobel Prize Scientist Joint, Research Center, East China University of Science and Technology, Shanghai, China; ^3^Department of Chemistry, University of Texas at Austin, Austin, TX, United States

**Keywords:** theranostic, peroxynitrite, coumarin, chemosensor, fluorescence

## Abstract

Two novel drug-conjugates based on a “coumarin linker” have been designed for the synergic release of a therapeutic agent and fluorescent probe for the potential application of theranostics. The drug conjugates; **CC-RNS** and **CI-RNS** were designed to be activated by reactive oxygen species or reactive nitrogen species (ROS/RNS). The fluorescence OFF-ON response was triggered by the peroxynitrite-mediated transformation of a boronic acid pinacol ester to a phenol moiety with simultaneous release of the therapeutic agents (Confirmed by HRMS). The limit of detection for peroxynitrite using **CC-RNS** and **CI-RNS** was 0.29 and 37.2 μM, respectively. Both **CC-RNS** and **CI-RNS** demonstrated the ability to visualize peroxynitrite production thus demonstrating the effectiveness of these probes for use as tools to monitor peroxynitrite-mediated drug release in cancer cell lines.

## Introduction

Theranostic systems, are the combination of a diagnostic and therapeutic and represent an emerging area with regard to effective cancer treatment. There have been an increasing number of examples of small-molecule theranostics which have good selectivity and high anti-tumor activity (Aulic et al., 2013; Kumar et al., 2015). In this respect, new theranostic probes are required for whole organism fluorescence tumor imaging and image guided surgery (Blau et al., 2018).

Real time monitoring of drug action is a prime concern for target specific cancer treatment. Redox-responsive chemotherapy is gaining attention. In such cases, a chemotherapeutic molecule is commonly attached to a fluorophore *via* a sacrificial self immolative linker (i.e., disulphide) (Wu et al., 2014; Gangopadhyay et al., 2017). Disulphide linkages are selectively cleaved in cancer cells by biological reducing agents, including glutathione (GSH); owing to high levels in cellular environments (2–10 mM) (Lu, 2013). Other examples of theranostic systems include hypoxia-induced and hydrogen peroxide activatable self-immolative systems (Kumar et al., 2014, 2016).

**Figure 1:**
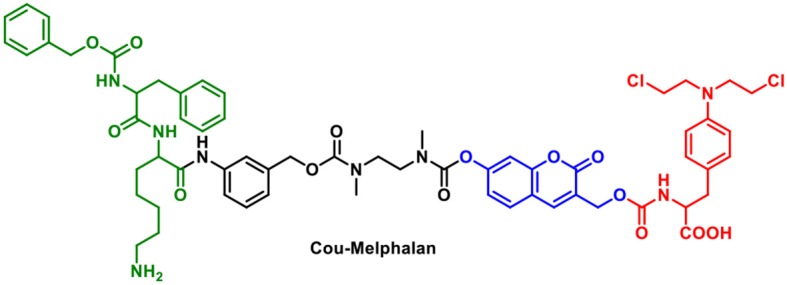
Structure of **Cou-Melphalan**.

A potential problem for “multi-component” systems is that the chromophore and therapy module could be released independently. One method to circumvent this problem is to use the fluorophore as the “linker” which can be functionalized with both an activating group and a therapeutic. In seminal work, Shabat et al. designed a theranostic prodrug using a self-immolative coumarin linker (Figure 1). The drug-delivery system uses a 7-hydroxycoumarin with a hydroxymethyl substituent. The phenolic alcohol of 7-hydroxycoumarin is linked to an activating group and the hydroxymethyl substituent acts as an attachment point for a drug or targeting group. In this example; **Cou-Melphalan** incorporates melphalan as a therapeutic with attachments *via* carbamates. This theranostic was designed to be activated by Cathespin B (Weinstain et al., 2013).

**Figure 2:**
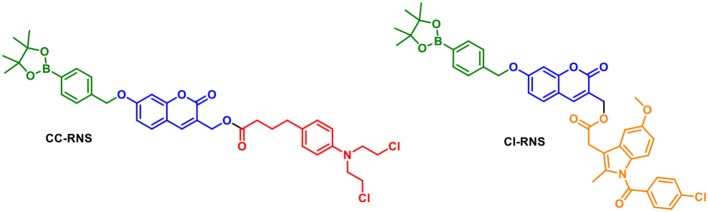
Structures of **CC-RNS** and **CI-RNS**.

**Scheme 1:**
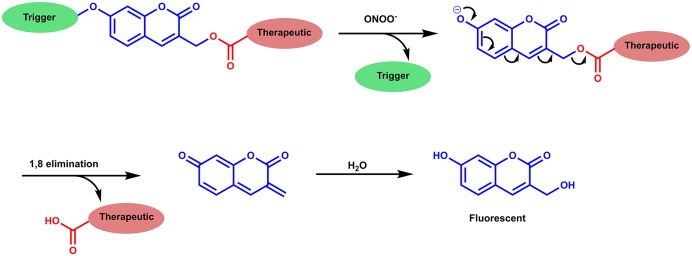
Proposed disassembly mechanism of **CC-RNS** and **CI-RNS**, adapted from Weinstain et al. (2013).

We were interested in utilizing the “coumarin linker” since they are natural products with simple preparative routes. Coumarin has important biological activities including; anti-tumor, anti-HIV and anti-bacterial properties, with multiple coumarin-derived molecules reported to cross the blood brain barrier (Borges et al., 2005; Yang et al., 2015). Various activating mechanisms for theranostics have been used; including activation by reactive oxygen species (ROS), intracellular thiols and enzymes (Chan et al., 2012). The reactive nitrogen species (RNS), peroxynitrite (ONOO^−^) is of interest due to its orthogonal reactivity with boronate esters and increased reactivity compared to its equivalent reactive oxygen species (ROS), hydrogen peroxide (H_2_O_2_) (Sedgwick et al., 2016, 2017; Wu et al., 2018). To this end, we set out to design ONOO^−^ activatable systems (Figure 2) (Yang et al., 2006; Sedgwick et al., 2016, 2018). The probes **CI-RNS** and **CC-RNS** were designed with ester linkers, in order to determine whether the same 1–8 self-immolative effect can occur, as in Shabat's system. Since, the use of an ester attachment provides access to probe-based systems using simpler synthetic procedures (Weinstain et al., 2013). The proposed disassembly mechanism is illustrated in Scheme 1.

*In vivo* ONOO^−^ is generated through the diffusion limited reaction of superoxide (O_2_^•−^) and nitric oxide (NO). Both O_2_^•−^ and NO are non-toxic *in vivo* due to efficient methods to minimize accumulation (Beckman, 1996). Under proinflammatory conditions, production of O_2_^•−^ and NO is activated, which inevitably increases the concentration of ONOO^−^ (Pacher et al., 2007). Typically, *in vitro* studies, the concentration of ONOO^−^ is enhanced by the ONOO^−^ donor SIN1 or stimulation of an inflammatory response using LPS and IFN-γ. The repurposing of the boronate-based probe peroxyresorufin-1 (PR1) Miller et al. (2005) to detect ONOO^−^ *in vitro* using these methods was reported by Weber et al. (2018). Interestingly, there is no reported data for loss of drug function through reaction with ONOO^−^. Examples of drug delivery systems which are activated by H_2_O_2_ have shown that, on release of the drug, the drug released still has the desired therapeutic response (Kumar et al., 2014; Wang et al., 2017).

As therapeutic payloads we chose; chlorambucil and indomethacin, providing distinct mechanisms of action for our theranostic probes. Chlorambucil is a DNA alkylating agent from the nitrogen mustard family. It is used to treat leukemia, Hodgkin's disease and non-Hodgkin lymphomas (Begleiter et al., 1996). Nitrogen mustards are generally unstable (Wang et al., 2010). Off-target reactions with proteins and biological thiols reduce drug potency, requiring higher dosages to produce a substantial therapeutic response. The repurposing and targeting of chlorambucil to enhance treatment scenarios are currently being investigated with successful preliminary studies in breast and pancreatic cancer cell lines (Millard et al., 2013; Kaur et al., 2017).

Indomethacin is primarily a non-steroidal anti-inflammatory drug (NSAID). Indomethacin inhibits the cyclooxygenase enzymes which catalyze the production of prostaglandins; responsible for inflammation and pain (Lucas, 2016). More recently; the effect of indomethacin on the modulation of the inflammatory responses in cancer have been investigated. Indomethacin has been shown to reduce cell migration, invasion, and metastasis in breast and colon cancer cell lines (Ackerstaff et al., 2007; Guo et al., 2013).

## Results and Discussion

**Scheme 2:**
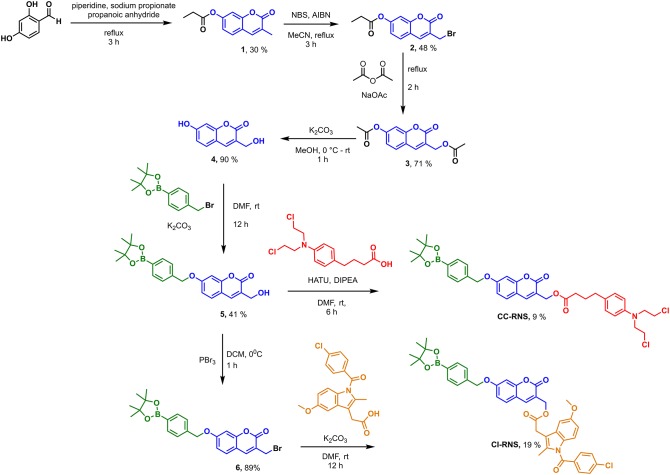
Synthesis scheme of **CI-RNS** and **CC-RNS**.

**CI-RNS** and **CC-RNS** were synthesized according to Scheme 2. **CI-RNS** was prepared over seven steps. **CC-RNS** was synthesized in six steps. First, coumarin-derivative (**4**) was obtained using previous literature procedures (Kim et al., 2014; Behara et al., 2017). Coumarin (**1**) was obtained by reacting 2,4-dihydroxybenzaldehyde with propionic anhydride in the presence of sodium propionate and piperidine. Compound **1** subsequently underwent radical bromination to afford compound **2**; which was transformed into compound **3** *via* acetylation. Compound **3** was deprotected with potassium carbonate in MeOH to yield coumarin (**4**). 4-(Bromomethyl)phenylboronic acid pinacol ester was added to compound **4** in an alkylation reaction to produce compound **5** in moderate yield. To generate **CI-RNS**; compound **5** underwent bromination in the presence of PBr_3_ to produce **6**. Thereafter, **CI-RNS** was obtained by reacting indomethacin in the presence of potassium carbonate, to afford the desired compound in 17% yield. To prepare **CC-RNS**; compound **5** was reacted with indomethacin using a HATU cross coupling reaction. This reaction gave the desired compound in a 9% yield.

**FIGURE 3:**
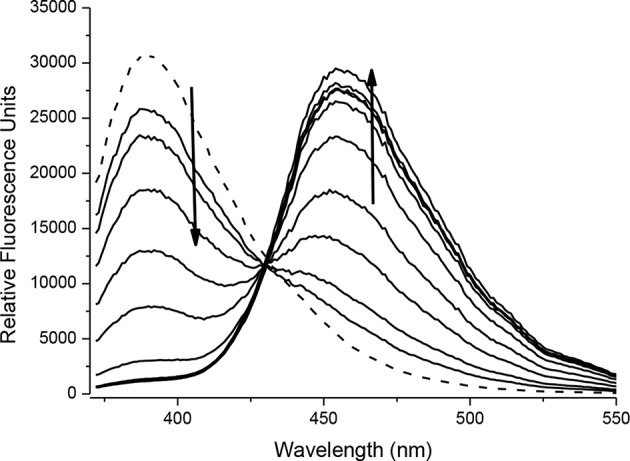
Fluorescence spectra of **5** (10 μM) in the presence of ONOO^−^ (0, 2, 4, 6, 8, 10, 12, 14, 16, 18, 20 μM). The data was collected in PBS buffer, pH = 7.3 at 25°C where λ_ex_ = 345 (16 bandwidth) nm. The dotted line represents probe only.

Intermediate compound **5** is an example of a ONOO^−^ activatable molecular probe. Therefore, the reactivity of **5** with ONOO^−^ was investigated. All measurements were recorded in a PBS buffer solution (pH = 7.3) at ambient temperature. The measurements were recorded instantly after ONOO^−^ addition. There is no change in UV-VIS absorption maximum at 315 nm on the addition of ONOO^−^ (20 μM) to the probe **5** (20 μM) (Supplementary Figure 1). The fluorescence spectra of probe **5** (10 μM), shows a ratiometric response. With increasing concentrations of ONOO^−^ (0–20 μM), there is a decrease in peak at 390 nm and emergence of a new peak at 460 nm; characteristic of free coumarin (Figure 3, Supplementary Figure 2). The limit of detection for **5** was calculated to be 76.5 nM (Supplementary Figure 3). The formation of free coumarin was also supported by mass spectroscopic studies; finding [M]^−^ = 192.0417 (Supplementary Figure 4).

**FIGURE 4:**
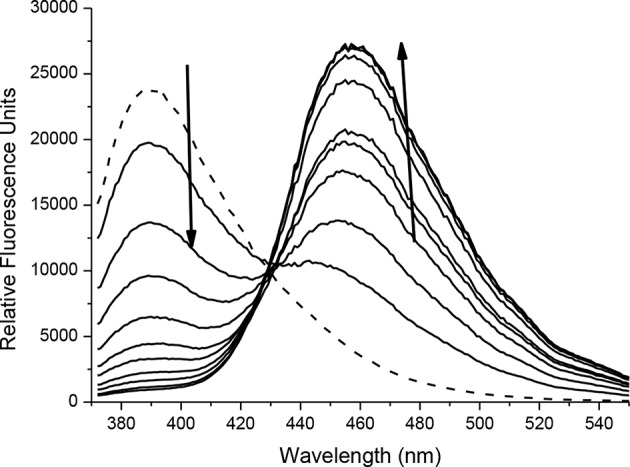
Fluorescence spectra of **6** (10 μM) in the presence of ONOO^−^ (0, 2, 4, 6, 8, 10, 12, 14, 16, 18, 20 μM). The data was collected in PBS buffer, pH = 7.3 at 25°C where λ_ex_ = 345 (16 bandwidth) nm. The dotted line represents probe only.

The spectroscopic properties of intermediate **6** were also investigated. 4-bromoumbelliferone shows weak fluorescence at 438 nm, therefore **6** is expected to be non-fluorescent (Thasnim and Bahulayan, 2017). This is due to the less electron-donating effect of the bromomethyl group compared to the methyl alcohol. Addition of ONOO^−^ results in formation of 3-(bromomethyl)-7-hydroxy-2*H*-chromen-2-one; this is confirmed by mass spectroscopic analysis; where exact mass for C_10_H_7_BrO_3_ [M]^−^ = 253.9597 finds [M]^−^ = 253.9569 (Supplementary Figure 5). There is no change in the UV-Vis response with absorption maxima at 340 nm (Supplementary Figure 6). Fluorescence studies carried out in PBS buffer pH = 7.3 at ambient temperature shows that **6** has a fluorescence emission at 390 nm. On addition of ONOO^−^ (0–20 μM) there is a decrease in emission at 390 nm and increase in a new emission peak at 455 nm (Figure 4, Supplementary Figure 7). The limit of detection for **6** was calculated to be 54.6 nM (Supplementary Figure 8).

**FIGURE 5:**
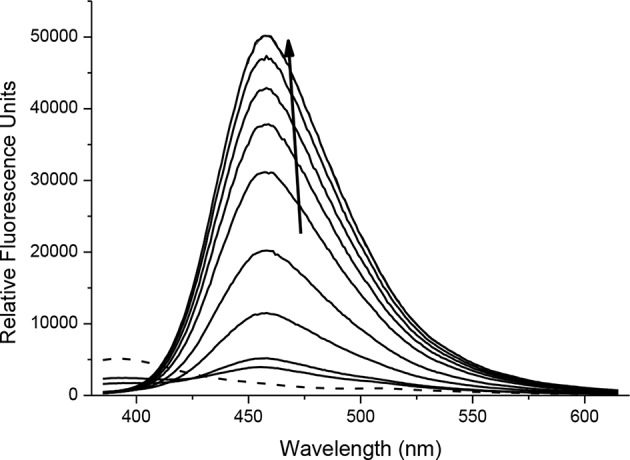
Fluorescence spectra of **CC-RNS** (10 μM) in the presence of ONOO^−^ (0–20 μM) in PBS buffer pH = 7.3. The data was collected at 25°C instantly after addition of peroxynitrite, where λ_ex_ = 345 (16 bandwidth) nm. The dotted line represents probe only.

**FIGURE 6:**
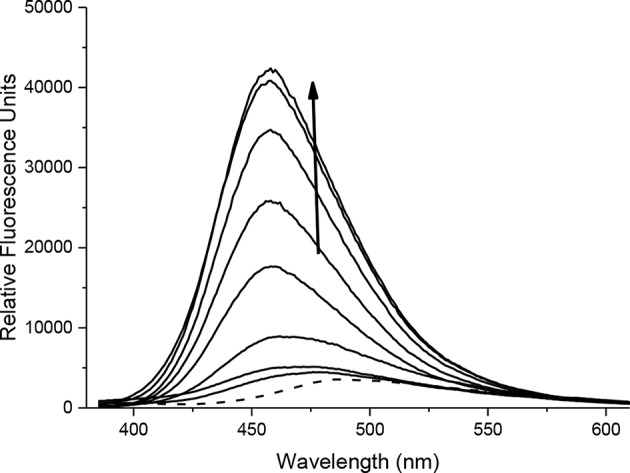
Fluorescence spectra of **CI-RNS** (10 μM) in the presence of ONOO^−^ (0, 10, 20, 25, 30, 35, 40, 45, 50 μM). The data was collected in PBS buffer, pH = 7.3 at 25°C where λ_ex_ = 345 (16 bandwidth) nm. The dotted line represents probe only.

With probes **CC-RNS** and **CI-RNS** in hand, spectroscopic evaluations were performed. All measurements were recorded in PBS buffer solution (pH = 7.3) at ambient temperature. The measurements were recorded instantly after ONOO^−^ addition. The UV-VIS spectrum of probe **CC-RNS (**20 μM) and **CI-RNS** (20 μM) were recorded with and without ONOO^−^ (50 μM—Supplementary Figures 9, 10). From the UV-VIS spectrum the probes have an initial absorption maximum at 315 nm. On addition of ONOO^−^, the absorption maximum shifts to 360 nm. The redox regulated response was monitored by subjecting 10 μM of each probe, **CC-RNS** and **CI-RNS**, to increasing concentrations of the biological oxidant ONOO^−^. Figures 5, 6 shows that initially both probes are non-fluorescent. There is a strong increase in emission at 460 nm for both probes; ca. **22-** fold for **CI-RNS** and **33-fold** for **CC-RNS** with increasing concentrations of ONOO^−^ (0–50 μM and 0–30 μM, respectively). The limit of detection (LOD) for **CC-RNS** is 0.29 μM and 37.2 μM for **CI-RNS** (Supplementary Figures 11–14).

The process of RNS triggered release of coumarin from **CC-RNS** and **CI-RNS** was supported by mass spectrometric evaluation. The experiments determined that [M]^−^ = 192.0438 and [M]^+^ = 192.0387 for **CC-RNS** and **CI-RNS**, respectively (Supplementary Figures 15, 16). This mass corresponds to formation of compound **4**; the expected cleavage product of the reaction. The formation of free coumarin demonstrates that these systems do release the drug payload *in situ*.

**FIGURE 7:**
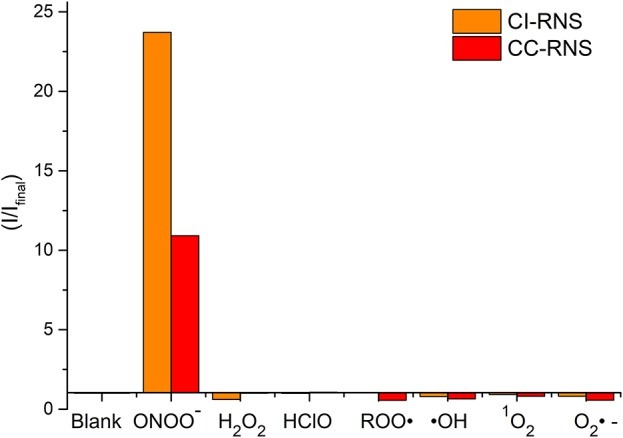
Selectivity data of **CC-RNS** and **CI-RNS** (10 μM) in the presence of ONOO^−^ (20 μM), H_2_O_2_ (200 μM), ClO^−^ (200 μM), ROO· (200 μM), OH (200 μM), O_2_^·−^ (200 μM), and ^1^O_2_ (200 μM) in PBS buffer pH = 7.3. The data was collected at 25°C after incubation for 15 min, where λ_ex_ = 345 (16 bandwidth) nm. Fluorescence intensity points were taken at λ_max_ = 460 nm.

**FIGURE 8:**
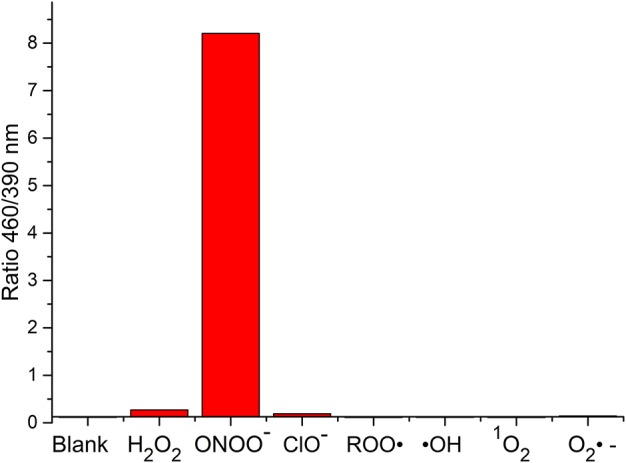
Selectivity data of **5** (10 μM) in the presence of ONOO^−^ (20 μM), H_2_O_2_ (100 μM), ClO^−^ (100 μM), ROO· (100 μM), OH (100 μM), O_2_^·−^ (100 μM), and ^1^O_2_ (100 μM) in PBS buffer pH = 7.3. The data was collected at 25°C after incubation for 15 min, where λ_ex_ = 345 (16 bandwidth) nm. Fluorescence intensity points were taken at λ_max_ = 460/390 nm.

**FIGURE 9:**
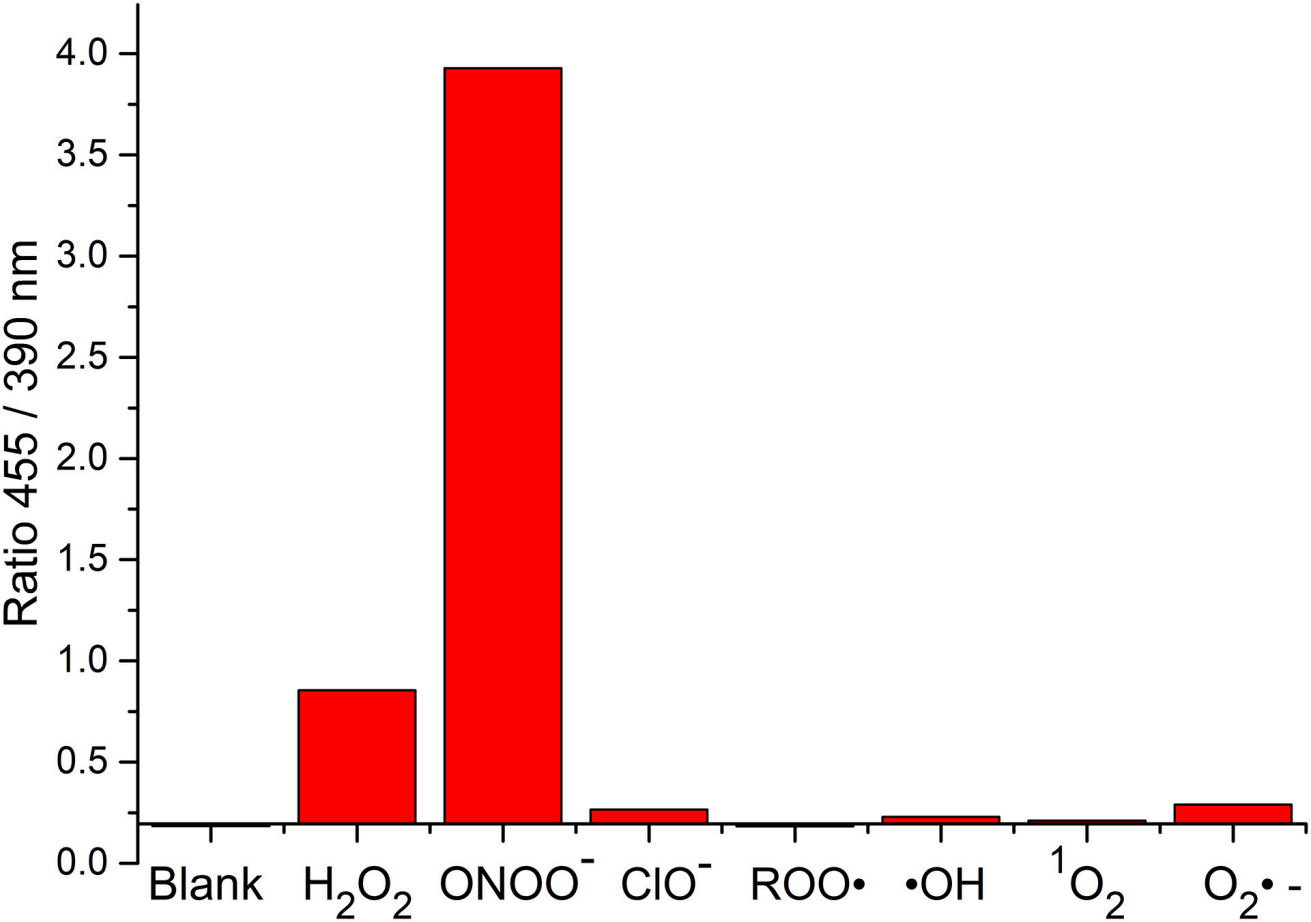
Selectivity data of **6** (10 μM) in the presence of ONOO^−^ (20 μM), H_2_O_2_ (100 μM), ClO^−^ (100 μM), ROO· (100 μM), ·OH (100 μM), O_2_^·−^ (100 μM), and ^1^*O*_2_ (100 μM) in PBS buffer pH = 7.3. The data was collected at 25°C after incubation for 15 min, where λ_ex_ = 345 (16 bandwidth) nm. Fluorescence intensity points were taken at λ_max_ = 455/390 nm.

The change in fluorescent properties of potential theranostics **CC-RNS**, **CI-RNS**, probes **5** and **6** were investigated in the presence of various biologically relevant ROS; hydrogen peroxide (H_2_*O*_2_), hypochlorite (ClO^−^), superoxide (O_2_^−•^), singlet oxygen (^1^*O*_2_), hydroxyl radical (HO•), and peroxyl radical (ROO•). Pleasingly, **CC-RNS**, **CI-RNS**, **5** and **6** are highly selective toward ONOO^−^ (Figures 7–9, Supplementary Figures 17–20).

**FIGURE 10:**
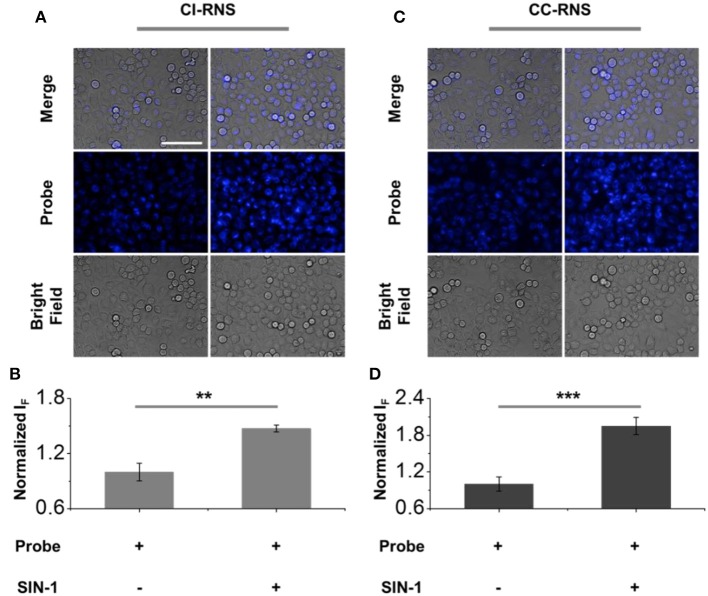
Fluorescence imaging and quantification of **(A,B) CI-RNS** (20 μM) and **(C,D) CC-RNS** (20 μM) in the presence of SIN-1 (500 μM) of HeLa cells. Excitation channel 360–400 nm, emission channel filtered = 410–480 nm. Scale bar = 100 μm. **P < 0.01; ***P < 0.001.

After confirming the selectivity and sensitivity of **CC-RNS** and **CI-RNS** for ONOO^−^, we evaluated the reaction of **CC-RNS** and **CI-RNS** with ONOO^−^ in cells. HeLa (cervical cancer) cells were pre-treated with **CI-RNS** or **CC-RNS**. Subsequently, SIN-1 (an ONOO^−^ donor) was added to produce intracellular ONOO^−^. As shown in Figure 10, the probe alone resulted in a weak fluorescence in cells. However, treatment with SIN-1 led to a significant increase in the fluorescence intensity enabling the visualization of ONOO^−^ in living cells. Cytotoxicity studies for both **CC-RNS** and **CI-RNS** indicate that the probes are almost non-toxic to Hela cells after incubation with SIN-1 and illumination (ESI- Supplementary Figures 21, 22). Hence, no further imaging studies were undertaken.

## Conclusion

In this work, we have developed two novel drug conjugate systems, **CC-RNS** and **CI-RNS**. The drug conjugate systems were designed to integrate a Shabat-based “coumarin linker” with an ONOO^−^ trigger. The systems incorporated the therapeutics drug payloads of chlorambucil and indomethacin as esters for potential theranostic application. Pleasingly, for each system *in solution*, the probes displayed a selective turn-on response toward ONOO^−^. In addition, *in vitro* evaluation with HeLa cells demonstrated that the probes were able to successfully visualize endogenous ONOO^−^ production, providing a potential platform to be able to monitor ONOO^−^ mediated drug release in cancer cell lines. We are currently working on the development of longer wavelength “fluorophore linkers” using ester attachments to appropriate therapeutic units that will facilitate the use of such probes in an animal model in order to evaluate their therapeutic value.

## Data Availability Statement

All datasets generated for this study are included in the article/Supplementary Material.

## Author Contributions

AS originated the idea and developed the synthetic route. JG and MO carried out synthesis. MO performed fluorescence experiments. H-HH carried out cell imaging studies under supervision from X-PH. MO wrote the manuscript with the support of SB and TJ. All authors read and approved the final manuscripts.

### Conflict of Interest

The authors declare that the research was conducted in the absence of any commercial or financial relationships that could be construed as a potential conflict of interest.
