# Supplementary material for: Peroxynitrite Activated Drug Conjugate Systems Based on a Coumarin Scaffold Toward the Application of Theranostics
Source: Front Chem. 2019 Dec 5;7:775. doi: 10.3389/fchem.2019.00775 (PMC6906548; doi:10.3389/fchem.2019.00775)
Supplement: Supplementary file 1 [file Table_1.docx]

Peroxynitrite activated drug conjugate systems based on a coumarin scaffold towards the application of theranostics.

Maria L. Odyniec^1*^, Hai-Hao Han,^3^ Jordan E. Gardiner^1^, Adam C. Sedgwick^,2^, Xiao-Peng He,^3^ Steven D. Bull,^1^ Tony D. James.^1^

^1^Department of Chemistry, University of Bath, Bath, BA2 7AY, U.K

^2^Department of Chemistry, University of Texas at Austin, 105 E, 24^th^ Street, A5300, Austin, USA.

^3^Key Laboratory for Advanced Materials & Feringa Nobel Prize Scientist Joint, Research Center, East China University of Science and Technology, 130 Meilong Rd., Shanghai 200237, P.R. China

Supplementary Material

1. **Synthesis …………………………………………………………………………………..**1
2. **NMR ………………………………………………………………………………………9**
3. **Preparation of ROS………………………………………………………………………25**
4. **Fluorescence and UV-VIS………………………………………………………………..26**
5. **Cell Culture……………………………………………………………………………….26**
6. **Fluorescence imaging in live cells………………………………………………………..26**
7. **Supplementary Data……………………………………………………………………...27**

**1 Synthesis**

**Synthesis of CC-RNS**

Supplementary Scheme 1 – Synthesis of CC-RNS

**Experimental**

**3-methyl-7-propionyl-2H-chromen-2-one (1**)

2,4-dihydroxybenzaldehyde (3.00 g, 21.7 mmol), sodium propionate (4.5 g, 46.8 mmol), piperidine (0.4 mL) and propanoic anhydride (7.54 mL) were added to together and heated to reflux for three hours. The resulting solution was poured into H_2_O at 0 ºC (100 mL) and the precipitate filtered. The precipitate was taken up in ethyl acetate (50 mL), washed with 1 M HCl (50 mL) and H_2_O (3 x 50 mL). The solution was dried (MgSO_4_) and concentrated *in vacuo* to afford the pure product as a cream solid (1.5 g, 4.64 mmol, 30 %). ^1^H NMR (500 MHz, CDCl_3_) δ 7.49 (d, *J* = 1.7 Hz, 1H, Ar-*H*), 7.40 (d, *J* = 8.4 Hz, 1H, Ar*-H*), 7.07 (d, *J* = 2.2 Hz, 1H, Ar-*H*), 7.00 (dd, *J* = 8.5, 2.2 Hz, 1H, Ar-*H*), 2.61 (q, *J* = 7.5 Hz, 2H, COC*H*_2_CH_3_), 2.19 (d, *J* = 1.4 Hz, 3H, R-CC*H*_3_), 1.27 (t, *J* = 7.5 Hz, 3H, RCH_2_C*H*_3_). ^13^C NMR (126 MHz, CDCl_3_) δ 172.38, 161.89, 153.68, 152.15, 138.69, 127.53, 125.15, 118.20, 117.30, 109.94, 27.70, 17.10, 8.90. IR (thin film) ν max (cm^-1^) 1760.87, 1712.73 (C=O). FTMS (p ESI): m/z calculated for C_13_H_12_O_4_ requires 233.080 for [M]^+^, found 233.0807. MP = 135-137 ºC

**3-(bromomethyl)-7-propionyl-2H-chromen-2-one (2)**

3-methyl-7-propionyl-2H-chromen-2-one (1.5 g, 6.46 mmol), NBS (2.3 g, 12.9 mmol) and AIBN (0.11 g, 0.65 mmol) were added to MeCN and heated to reflux. After three hours, the reaction was cooled and concentrated *in vacuo*. Following this the reaction was diluted with DCM (50 mL) and washed with NaHCO_3_ (50 mL), H_2_O (3 x 25 mL) and brine (50 mL). The solution was dried (MgSO_4_) and solvent removed *in vacuo* to afford the product as a white solid (0.8 g, 2.5 mmol, 40 %). The product was taken on to the next step. ^1^H NMR (500 MHz, CDCl_3_) δ 7.84 (d, *J* = 0.8 Hz, 1H, Ar*-H*), 7.51 (d, *J* = 8.4 Hz, 1H, Ar*-H*), 7.14 (d, *J* = 2.3 Hz, 1H, Ar*-H*), 7.08 (dd, *J* = 8.5, 2.2 Hz, 1H, Ar*-H*), 4.42 (d, *J* = 0.8 Hz, 2H, RC*H*_2_-Br), 2.63 (q, *J* = 7.5 Hz, 2H, RC*H*_2_CH_3_), 1.28 (t, *J* = 7.5 Hz, 3H, RCH_2_C*H*_3_). ^13^C NMR (126 MHz, CDCl_3_) δ 159.55, 154.33, 153.53, 141.32, 128.69, 124.84, 118.74, 116.56, 110.25, 27.73, 27.42, 25.16, 8.88. IR (thin film) ν max (cm^-1^) 1761.92, 1722.78 (C=O). FTMS (p ESI): m/z calculated for C_13_H_11_O_4_Br requires 332.9733 for [M]^+^, found 332.9737. MP = 118-120 ºC

**(7-acetyl-2-oxo-2H-chromen-3-yl)methyl acetate (3)**

3-(bromomethyl)-7-propionyl-2H-chromen-2-one (0.8 g, 2.5 mmol) and NaOAc (0.62 g, 7.5 mmol) were added to acetic anhydride (10 mL). The reaction was heated to reflux for two hours. On completion of the reaction by TLC the reaction was cooled to 100 ºC and filtered hot to remove salts. Residual acetic anhydride was removed *in vacuo* and azeotrope with toluene to afford the final compound as a brown solid (0.6 g, 2.17 mmol, 71 %). ^1^H NMR (500 MHz, CDCl_3_) δ 7.74 (q, *J* = 1.0 Hz, 1H, Ar*-H*), 7.50 (d, *J* = 8.5 Hz, 1H, Ar-*H*), 7.13 (d, *J* = 2.2 Hz, 1H, Ar-*H*), 7.07 (dd, *J* = 8.5, 2.2 Hz, 1H, Ar*-H*), 5.06 (d, *J* = 1.2 Hz, 2H, R-C*H*_2_COCH_3_), 2.34 (s, 3H, R-COC*H*_3_), 2.15 (s, 3H, R-CH_2_COC*H*_3_). ^13^C NMR (126 MHz, CDCl_3_) δ 170.49, 168.61, 159.88, 154.09, 153.12, 140.23, 128.69, 123.06, 118.59, 116.53, 110.21, 61.08, 21.10, 20.86. IR (thin film) ν max (cm^-1^) 1758.51, 1741.63, 1712.10 (C=O). FTMS (p ESI): m/z calculated for C_14_H_12_O_6_ requires 299.0526 for [M]^+^ , found 299.0558. MP = 140-142 ºC

**7-hydroxy-3-(hydroxymethyl)-2H-chromen-2-one (4)**

(7-acetyl-2-oxo-2H-chromen-3-yl)methyl acetate (1.5 g, 5.4 mmol) was dissolved in MeOH (60 mL) and cooled to 0 ºC. K_2_CO_3_ (2.3 g, 16.3 mmol) was added and the reaction was left for 1 h. The reaction solvent was removed *in vacuo* and diluted with H_2_O (20 mL). The resulting solution was acidified to pH 3 and the pure product precipitated as a brown solid (1.1 g, 5.7 mmol, 90 %). ^1^H NMR (500 MHz, DMSO-*d*_6_) δ 10.46 (s, 1H), 7.83 (s, 1H, Ar*-H*), 7.54 (d, *J* = 8.4 Hz, 1H, Ar*-H*), 6.77 (dd, *J* = 8.5, 2.3 Hz, 1H, Ar*-H*), 6.71 (d, *J* = 2.3 Hz, 1H, Ar-*H*), 5.31 (s, 1H, R-O*H*), 4.29 (s, 2H, RC*H*_2_-OH). ^13^C NMR (126 MHz, DMSO-*d*_6_) δ 160.94, 160.61, 154.71, 138.38, 129.73, 124.72, 113.54, 111.94, 102.30, 58.57. IR (thin film) ν max (cm^-1^) 3417.87 (O-H). FTMS (p ESI): m/z calculated for C_10_H_8_O_4_ requires 215.0315 for [M]^+^, found 215.0307. MP = 201-203 ºC

**3-(hydroxymethyl)-7-((4-(4,4,5,5-tetramethyl-1,3,2-dioxaborolan-2-yl)benzyl)oxy)-2H-chromen-2-one (5)**

7-hydroxy-3-(hydroxymethyl)-2H-chromen-2-one (0.35 g, 1.82 mmol), 2-(4-(bromomethyl)phenyl)-4,4,5,5-tetramethyl-1,3,2-dioxaborolane (0.56 g, 1.91 mmol) and K_2_CO_3_ (0.50 g, 3.64 mmol) were added to DMF (5 mL) and left to stir overnight. The resulting solution was diluted with EtOAc (25 mL), washed with H_2_O (3 x 50 mL) and brine (2 x 20 mL), dried (MgSO_4_) and concentrated *in vacuo*. The final product was purified using column chromatography (40:60, EtOAc/Pet ether) to afford a white solid (0.246 g, 0.74 mmol, 41 %). ^1^H NMR (500 MHz, CDCl_3_) δ 7.86 – 7.82 (m, 2H, Ar*-H*), 7.66 (d, *J* = 1.4 Hz, 1H, Ar*H*), 7.44 – 7.41 (m, 2H, Ar*-H*), 7.39 (d, *J* = 8.6 Hz, 1H, Ar-*H*), 6.92 (dd, *J* = 8.6, 2.4 Hz, 1H, Ar*-H*), 6.88 (d, *J* = 2.4 Hz, 1H, Ar-*H*), 5.15 (s, 2H, ROC*H*_2_CR), 4.58 (d, *J* = 1.2 Hz, 2H, ROC*H*_2_CR), 1.34 (s, 12H, Bpin). ^13^C NMR (126 MHz, CDCl_3_) δ 161.68, 161.49, 154.92, 138.92, 138.77, 135.16, 128.79, 126.54, 124.38, 113.46, 112.85, 101.79, 83.89, 70.41, 61.32, 24.85. FTMS (p ESI): m/z calculated for C_23_H_25_O_6_B requires 431.1640 for [M]^+^, found 431.1641. IR (thin film) ν max (cm^-1^): 3407.94 (O-H), 1698.17 (C=O). MP = 198-200 ºC

**(2-oxo-7-((4-(4,4,5,5-tetramethyl-1,3,2-dioxaborolan-2-yl)benzyl)oxy)-2H-chromen-3-yl)methyl 4-(4-(bis(2-chloroethyl)amino)phenyl)butanoate (CC-RNS)**

Chlorambucil (0.11 g, 0.36 mmol) and HATU (0.13 g, 0.36 mmol) were added to DMF (2 mL) and stirred for 20 mins. A mixture of 3-(hydroxymethyl)-7-((4-(4,4,5,5-tetramethyl-1,3,2-dioxaborolan-2-yl)benzyl)oxy)-2H-chromen-2-one (0.11 g, 0.33 mmol) and DIPEA (0.12 mL) in DMF (1 mL) was added to the stirring solution, dropwise. After 6 hours, the reaction was diluted with EtOAc (20 mL) and washed with H_2_O (3 x 50 mL) and brine (3 x 20 mL), dried (MgSO_4_) and concentrated in vacuo. The pure product was isolated with column chromatography (20:80 – EtOAc: pet ether) to afford the pure product as a cream oil (20 mg, 0.03 mmol, 9 %). ^1^H NMR (500 MHz, CDCl3) δ 7.84 (dd, *J* = 7.9, 1.8 Hz, 1H, Ar*-H*), 7.69 (s, 1H, Ar*-H*), 7.40 (ddd, *J* = 23.4, 8.3, 1.8 Hz, 2H, Ar*-H*), 7.10 – 7.03 (m, 1H, Ar*-H*), 6.97 – 6.86 (m, 1H, Ar*-H*), 6.67 – 6.59 (m, 1H, Ar*-H*), 5.15 (d, *J* = 1.8 Hz, 1H, O-C*H*_2_-R), 5.03 (s, 1H, O-C*H*_2_-R), 3.74 – 3.67 (m, 2H, N-C*H_2_*CH_2_Cl), 3.67 – 3.57 (m, 2H, N-*CH_2_CH_2_*Cl), 2.57 (t, *J* = 7.8 Hz, 2H, Ar-*CH_2_*CH_2_), 2.40 (td, *J* = 7.4, 1.8 Hz, 2H, COC*H*_2_CH_2_), 1.93 (q, *J* = 7.5 Hz, 2H, , Ar-CH_2_C*H*_2_ CH_2_CO), 1.39 – 1.22 (m, 12H, Bpin). ^13^C NMR (126 MHz, CDCl_3_) δ 173.19, 161.88, 160.67, 155.36, 141.53, 138.70, 135.17, 129.73, 129.72, 129.00, 126.54, 120.08, 113.48, 112.57, 101.78, 83.89, 70.43, 61.28, 53.78, 40.35, 33.93, 33.86, 33.47, 32.91, 31.91, 29.68, 29.64, 29.35, 26.60, 26.45, 24.85, 22.68, 14.11. IR (thin film) ν max (cm^-1^) 1731.25 (C=O). FTMS (p ESI): m/z calculated for C_37_H_42_O_7_Cl_2_BN requires 716.2329 for [M]^+^, found 716.2334.

**Synthesis of CI-RNS**

**Supplementary Scheme 2**- Synthesis of **CI-RNS**
 **Experimental**

**3-(bromomethyl)-7-((4-(4,4,5,5-tetramethyl-1,3,2-dioxaborolan-2-yl)benzyl)oxy)-2*H*-chromen-2-one (6)**

7-hydroxy-3-(hydroxymethyl)-2H-chromen-2-one (0.093 g, 0.23 mmol), dissolved in DCM (3 mL) was added to PBr_3_ (0.074 g, 0.28 mmol) in DCM (3 mL) at -5 ºC and stirred for 1 hour. On completion of the reaction, the mixture was diluted with DCM (25 mL), washed with H_2_O (3 x 50 mL), brine (2 x 20 mL) and Na_2_S_2_O_3_ (100 mL). The resulting solution was dried (MgSO_4_) and concentrated *in vacuo*. The oil was azeotrope with chloroform and concentrated *in vacuo* to afford the product, a yellow oil. (0.11 g, 0.2288 mmol, 89 %). ^1^H NMR (500 MHz, CDCl_3_) δ 7.84 (d, *J* = 8.1 Hz, 2H, Ar-*H*), 7.78 (d, *J* = 0.7 Hz, Ar-*H*), 7.43 (d, *J* = 8.2 Hz, 2H, Ar-*H*), 7.39 (d, *J* = 8.6 Hz, 1H, Ar-*H*), 6.93 (dd, *J* = 8.6, 2.4 Hz, 1H, Ar-*H*), 6.88 (d, *J* = 2.5 Hz, 1H, Ar-*H*), 5.16 (s, 2H, ROC*H*_2_CR), 4.42 (d, *J* = 0.8 Hz, 2H, RC*H*_2_Br ), 1.35 (s, 12H, Bpin). ^13^C NMR (126 MHz, CDCl_3_) δ 161.90, 160.2, 156.91, 143.7, 136.71, 133.15, 130.4, 126.64, 127.3, 112.85, 111.51, 104, 84.90, 70.81, 34.7, 24.60. IR (thin film) ν max (cm^-1^) 2985.06 (C-H), 1711.87 (C=O), 1612.58 (C=C). FTMS (p ESI): m/z calculated for C_23_H_24_BBrO_5_ requires 469.08219 for [M]^+^, found 470.0610.

**(2-oxo-7-((4-(4,4,5,5-tetramethyl-1,3,2-dioxaborolan-2-yl)benzyl)oxy)-2H-chromen-3-yl)methyl 2-(1-(4-chlorobenzoyl)-5-methoxy-2-methyl-1H-indol-3-yl)acetate - (CI-RNS)**

Indomethacin (0.087 g, 0.24 mmol) and K_2_CO_3_ (0.1 g, 0.73 mmol) were added to DMF (5 mL) and 3-(bromomethyl)-7-((4-(4,4,5,5-tetramethyl-1,3,2-dioxaborolan-2-yl)benzyl)oxy)-2*H*-chromen-2-one (0.11 g, 0.24 mmol) was added. The reaction was stirred at rt overnight. After which, the reaction was diluted with diluted with EtOAc (20 mL) and washed with water (3 x 50 mL) and brine (3 x 20 mL), dried (MgSO_4_) and concentrated *in vacuo* to afford the title compound as a pale-yellow oil (0.030 g, 17 %, 0.040 mmol). ^1^H NMR (500 MHz, CDCl_3_) δ 7.87 – 7.82 (m, 2H, Ar-*H*), 7.67 – 7.63 (m, 2H, Ar-*H*), 7.48 – 7.40 (m, 5H, Ar-*H*), 7.16 (d, *J* = 8.6 Hz, 1H, Ar-*H*), 6.98 (d, *J* = 2.5 Hz, 1H, Ar-*H*), 6.92 (d, *J* = 9.0 Hz, 1H, Ar-*H*), 6.89 (dd, *J* = 8.6, 2.4 Hz, 1H, Ar-*H*), 6.84 (d, *J* = 2.4 Hz, 1H, Ar-*H*), 6.69 (dd, *J* = 9.0, 2.6 Hz, 1H, Ar-*H*), 5.14 (s, 2H, RO-C*H*_2_-Ar), 5.06 (d, *J* = 1.1 Hz, 2H, RC-C*H*_2_COR), 3.76 (s, 2H, RO-C*H*_3_), 2.38 (s, 3H, RNC(C*H*_3_)), 1.35 (s, 12H, Bpin). ^13^C NMR (126 MHz, CDCl_3_) δ 170.42, 168.29, 161.87, 160.49, 156.09, 155.22, 141.13, 139.32, 138.71, 136.03, 135.18, 133.80, 131.17, 130.82, 130.49, 129.13, 129.10, 128.98, 126.57, 126.54, 119.72, 115.03, 113.46, 112.45, 112.27, 111.78, 101.72, 101.30, 83.91, 70.42, 61.76, 55.68, 30.37, 24.86. FTMS (p ESI): m/z calculated for C_42_H_39_BClNO_3_ requires 650.2723 for [M]^+^, found 650.2748. IR (thin film) ν max (cm^-1^) 2977.38 (C-H), 1722.89, 1698.33, 1608.01 (C=O).

**2 NMR**

**3-methyl-7-propionyl-2H-chromen-2-one (1)** – (500 MHz, CDCl_3_)

**3-methyl-7-propionyl-2H-chromen-2-one (1)**– (126 MHz, CDCl_3_)

**3-(bromomethyl)-7-propionyl-2H-chromen-2-one (2)**- (500 MHz, CDCl_3_)

**3-(bromomethyl)-7-propionyl-2H-chromen-2-one (2)-** (126 MHz, CDCl_3_)

**(7-acetyl-2-oxo-2H-chromen-3-yl)methyl acetate (3)** - (500 MHz, CDCl_3_)

**(7-**

**(7-acetyl-2-oxo-2H-chromen-3-yl)methyl acetate (3)- (**126 MHz, CDCl_3_)

**7-Hydroxy-3-(hydroxymethyl)-2H-chromen-2-one (4)–** (500 MHz, DMSO-*d*_6_)

**7-hydroxy-3-(hydroxymethyl)-2H-chromen-2-one (4) –** (126 MHz, DMSO-*d*_6_)

**3-(hydroxymethyl)-7-((4-(4,4,5,5-tetramethyl-1,3,2-dioxaborolan-2-yl)benzyl)oxy)-2H-chromen-2-one (5)–**(500 MHz, CDCl_3_).

**(hydroxymethyl)-7-((4-(4,4,5,5-tetramethyl-1,3,2-dioxaborolan-2-yl)benzyl)oxy)-2H-chromen-2-one (5)–**(126 MHz, CDCl_3_).

**(2-oxo-7-((4-(4,4,5,5-tetramethyl-1,3,2-dioxaborolan-2-yl)benzyl)oxy)-2H-chromen-3-yl)methyl 4-(4-(bis(2-chloroethyl)amino)phenyl)butanoate (CC-RNS)-** (500 MHz, CDCl_3_)

**(2-oxo-7-((4-(4,4,5,5-tetramethyl-1,3,2-dioxaborolan-2-yl)benzyl)oxy)-2H-chromen-3-yl)methyl 4-(4-(bis(2-chloroethyl)amino)phenyl)butanoate (CC-RNS)** (126 MHz, CDCl_3_)

**3-(bromomethyl)-7-((4-(4,4,5,5-tetramethyl-1,3,2-dioxaborolan-2-yl)benzyl)oxy)-2*H*-chromen-2-one (6)**- (500 MHz, CDCl_3_)**.**

**3-(bromomethyl)-7-((4-(4,4,5,5-tetramethyl-1,3,2-dioxaborolan-2-yl)benzyl)oxy)-2*H*-chromen-2-one (6)**- (126 MHz, CDCl_3_)

**(2-oxo-7-((4-(4,4,5,5-tetramethyl-1,3,2-dioxaborolan-2-yl)benzyl)oxy)-2H-chromen-3-yl)methyl 2-(1-(4-chlorobenzoyl)-5-methoxy-2-methyl-1H-indol-3-yl)acetate (CI-RNS) -** (500 MHz, CDCl_3_)

**(2-oxo-7-((4-(4,4,5,5-tetramethyl-1,3,2-dioxaborolan-2-yl)benzyl)oxy)-2H-chromen-3-yl)methyl 2-(1-(4-chlorobenzoyl)-5-methoxy-2-methyl-1H-indol-3-yl)acetate (CI-RNS)**- (126 MHz, CDCl_3_)

**3 Preparation of ROS**

**ROO•**

ROO**•** was generated from 2,2'-azobis (2-amidinopropane) dihydrochloride. AAPH (2, 2’ azobis (2- amidinopropane) dihydrochloride (0.1M) was added into deionized water, and then stirred at 37 °C for 30 min.

**O_2_^-^**

Superoxide was generated from KO_2_.
KO_2_ (1eq) and 18-crown-6 ether (2.5 eq) was dissolved in DMSO to afford a 0.1 M solution.

**•HO**

Hydroxyl radical was generated by the Fenton reaction.
To prepare •OH solution, hydrogen peroxide (H_2_O_2_, 10 eq) was added to Fe(ClO_4_)_2_ (1 eq) in de-ionised water to give a 0.1 M solution.

**ONOO^-^**

Simultaneously, 0.6 M KNO_2_, 0.6 M HC1, 0.7 M H_2_O_2_ were added to a 3 M NaOH solution at 0 ºC. The concentration of peroxynitrite was estimated by using extinction co-efficient of 1670 cm^-1^M^-1^ at 302 nm in 0.5 M sodium hydroxide solution.

**-OCl**

Hypochlorite is diluted into H_2_O (50 µL in 10 mL). The concentration of –OCl was determined from the absorption at 292 nm (Ɛ = 350 cm^-1^M^-1^).

**H_2_O_2_**

H_2_O_2_ is diluted into H_2_O (100 µL in 10 mL). The concentration of H_2_O_2_ was determined from the absorption at 240 nm (Ɛ = 43.6 cm^-1^M^-1^).

**4 Fluorescence and UV-VIS**

Fluorescence measurements were performed on Clariostar Monochromator Microplate Reader, utilising black F-bottom Greiner 96 Well Polystyrene Microplate. UV-VIS measurements were taken using clear F-bottom Greiner 96 Well Polystyrene Microplates. The data was collected using the MARS software package and processed in excel. All solvents used in fluorescence measurements were HPLC or fluorescence grade and water was de-ionised.

**5 Cell Culture**

Hela cells were maintained in a Dulbecco’s Modified Eagle’s Medium (Invitrogen, Carlsbad, CA, USA) supplemented with 10% fetal bovine serum (Gibco, Gland Island, NY, USA) in a humidified atmosphere of 5% CO_2_ and 95% air at 37℃ and split when the cells reached 90% confluency.

**6 Fluorescence imaging in live cells**

Cells were seeded on a black 96-well microplate with optically clear bottom (Greiner bio-one, Germany) overnight. To detect ONOO^-^, the cells were first incubated with **CI-RNS** or **CC-RNS** (20 μM, 1% DMSO in PBS, pH 7.4) for 30 min, followed by incubation with SIN-1 (500 μM) for 30 min. Then, cells were washed with PBS (phosphate buffered saline) three times. The fluorescence images were recorded using an Operetta high-content imaging system (Perkinelmer, US) and quantified by Columbus analysis system (Perkinelmer, US).

**7 Supplementary Data**

**Supplementary Figure 1**- UV-VIS of **5** (20 µM) in the presence of ONOO^-^ (20 µM). The data was collected in PBS buffer, pH = 7.3 at 25 º C.

**Supplementary Figure 2**- Dose dependence curve of **5** (10 µM) in the presence of ONOO^-^ (0, 2, 4, 6, 8, 10, 12, 14, 16, 18, 20 µM). The data was collected in PBS buffer, pH = 7.3 at 25 º C where λ_ex_= 345 (16 bandwidth) nm.

Supplementary Figure 3- Plots of relative fluorescence intensity of 5 (10 µM) as a function of ONOO^-^ concentration (as a ratio of the fluorescence intensity at λ_max_= 39 and 60 nm). Fluorescence studies were carried out in PBS buffer pH = 7.3 at 25 ºC, λ_ex_=345 (16 bandwidth) nm. Calculation for LOD = limit of detection (3σ/k) – of the linear section of the graph.


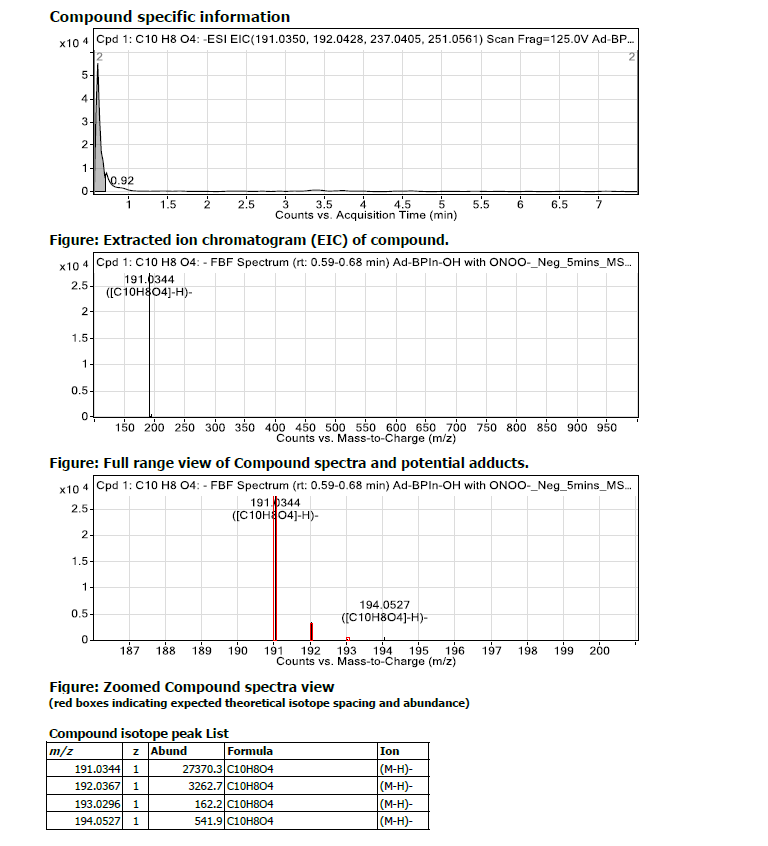


Supplementary Figure 4- HMRS of reaction of 5 with ONOO^-^ to generate free coumarin (4).


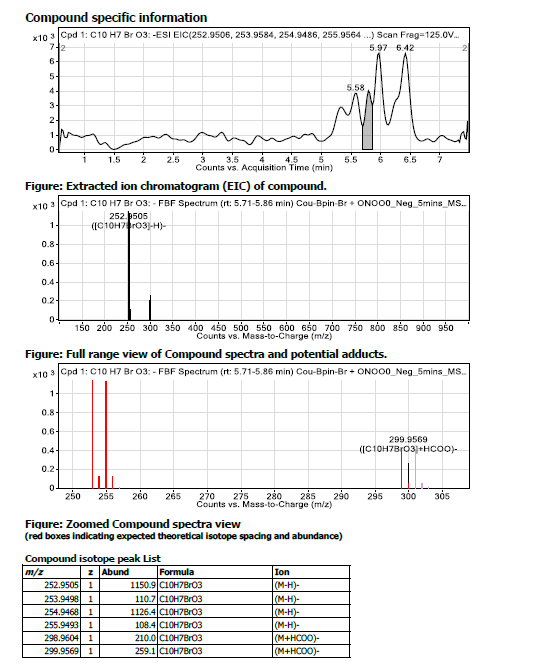


Supplementary Figure 5 - HMRS of reaction of 6 with ONOO^-^ to generate free Coumarin-Br.

**Supplementary Figure 6**- UV-VIS of **6** (20 µM) in the presence of ONOO^-^ (20 µM). The data was collected in PBS buffer, pH = 7.3 at 25 º C.

Supplementary Figure 7 - Dose dependence curve of 6 (10 µM) in the presence of ONOO^-^ (0-20 µM) in PBS buffer pH = 7.3. The data was collected at 25 º C instantly after addition of ONOO^-^, where λ_ex_= 345 (16 bandwidth) nm. Fluorescence intensity points were taken at λ_max_= 390 and 455 nm.

Supplementary Figure 8 - Plots of relative fluorescence intensity of 6 (10 µM) as a function of ONOO^-^ concentration (as a ratio of the fluorescence intensity at λ_max_= 390 and 455 nm). Fluorescence studies were carried out in PBS buffer pH = 7.3 at 25 ºC, λ_ex_=345 (16 bandwidth) nm. Calculation for LOD = limit of detection (3σ/k) – of the linear section of the graph.

**Supplementary Figure 9** *-* UV-Vis of **CC-RNS** (20 µM) in the presence of ONOO^-^ (50 µM) in PBS buffer pH = 7.3. The data was collected at 25 º C instantly after addition of ONOO^-^.

**Supplementary Figure 10** - UV-Vis spectra of **CI-RNS** (20 µM) with and without ONOO^-^ (50 µM – red line). The data was collected in PBS buffer, pH = 7.3 at 25 º C.

Supplementary Figure 11- Dose dependence curve of CC-RNS (10 µM) in the presence of ONOO^-^ (0-20 µM) in PBS buffer pH = 7.3. The data was collected at 25 º C instantly after addition of ONOO^-^, where λ_ex_= 345 (16 bandwidth) nm. Fluorescence intensity points were taken at λ_max_= 460 nm.

Supplementary Figure 12- Plots of relative fluorescence intensity of CC-RNS (10 µM) as a function of ONOO^-^ concentration (where I_final_ and I is the fluorescence intensity of the system at λ_max_= 460 nm as a function of final fluorescence response). Fluorescence studies were carried out in PBS buffer pH = 7.3 at 25 ºC, λ_ex_=345 (16 bandwidth) nm. Calculation for LOD = limit of detection (3σ/k) – of the linear section of the graph.

Supplementary Figure 13- Dose dependence curve of CI-RNS (10 µM) in the presence of ONOO^-^ (0-50 µM) in PBS buffer pH = 7.3. The data was collected at 25 º C instantly after addition of ONOO^-^, where λ_ex_= 345 (16 bandwidth) nm. Fluorescence intensity points were taken at λ_max_= 455 nm.

Supplementary Figure 14. - Plots of relative fluorescence intensity of CI-RNS (250 nM) a function of ONOO^-^ concentration (where I_final_ and I is the fluorescence intensity of the system at λ_max_= 460 nm as a function of final fluorescence response). Fluorescence studies were carried out in PBS buffer pH = 7.3 at 25 ºC, λ_ex_=345 (16 bandwidth) nm. Calculation for LOD = limit of detection (3σ/k) – of the linear section of the graph.


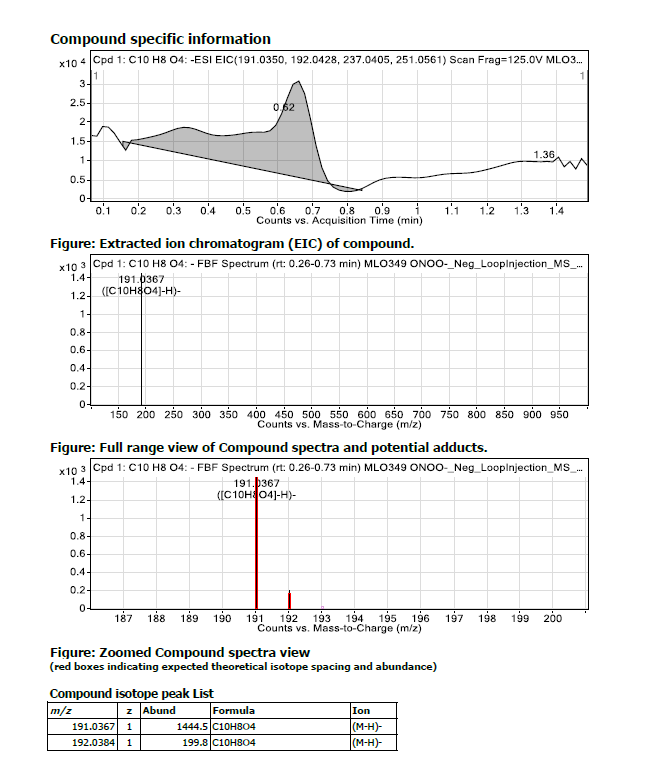


Supplementary Figure 15- HMRS of reaction of CC-RNS with ONOO^-^ to generate 4.


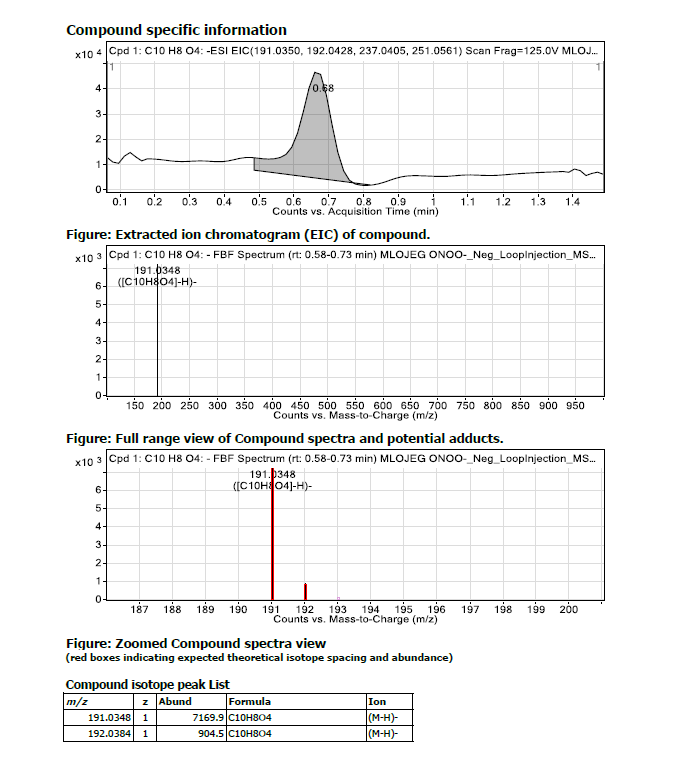


Supplementary Figure 16- HMRS of reaction of CI-RNS with ONOO^-^ to generate 4.

Supplementary Figure 17 – Fluorescence spectra of CC-RNS (10 µM) in the presence of ONOO^-^ (20 µM), H_2_O_2_ (200 µM), ClO^-^ (200 µM), ROO· (200 µM), ·OH (200 µM), O_2_^-•^ (200 µM), and ^1^O_2_ (200 µM) in PBS buffer pH = 7.3. The data was collected at 25 ºC after incubation for 15 mins, where λ_ex_= 345 (16 bandwidth) nm. The solid black line represents sensor only.

Supplementary Figure 18 – Fluorescence spectra of CI-RNS (10 µM) in the presence of ONOO^-^ (200 µM), H_2_O_2_ (200 µM), ClO^-^ (200 µM), ROO· (200 µM), ·OH (200 µM), O_2_^-•^ (200 µM), and ^1^O_2_ (200 µM) in PBS buffer pH = 7.3. The data was collected at 25 º C after incubation for 15 mins, where λ_ex_= 345(16 bandwidth) nm. The solid black line represents sensor only.

Supplementary Figure 19- Selectivity data of 5 (10 µM) in the presence of ONOO^-^ (20 µM), H_2_O_2_ (100 µM), ClO^-^ (100 µM), ROO· (100 µM), ·OH (100 µM), O_2_^-•^ (100 µM), and ^1^O_2_ (100 µM) in PBS buffer pH = 7.3. The data was collected at 25 º C after incubation for 15 mins, where λ_ex_= 345 (16 bandwidth) nm.

Supplementary Figure 20 - Selectivity data of 6 (10 µM) in the presence of ONOO^-^ (20 µM), H_2_O_2_ (100 µM), ClO^-^ (100 µM), ROO· (100 µM), ·OH (100 µM), O_2_^-•^ (100 µM), and ^1^O_2_ (100) µM in PBS buffer pH = 7.3. The data was collected at 25 º C after incubation for 15 mins, where λ_ex_= 345 (16 bandwidth) nm.

Supplementary Figure 21 – Cytotoxicity studies CC-RNS (0-20 µM), in HeLa cells. The probe was incubated with SIN-1 (1 mM) for 6 hrs followed by UV irradiation (365 nm) for 20 mins. Cells were left for further incubation for 24 hrs.

Supplementary Figure 22– Cytotoxicity studies CI-RNS (0-20 µM), in HeLa cells. The probe was incubated with SIN-1 (1 mM) for 6 hrs followed by UV irradiation (365 nm) for 20 mins. Cells were left for further incubation for 24 hrs.
